# The Neutralizing Antibody Response Elicited by Tembusu Virus Is Affected Dramatically by a Single Mutation in the Stem Region of the Envelope Protein

**DOI:** 10.3389/fmicb.2020.585194

**Published:** 2020-10-22

**Authors:** Junfeng Lv, Xiaoxiao Liu, Shulin Cui, Lixin Yang, Shenghua Qu, Runze Meng, Baolin Yang, Chonglun Feng, Xiaoyan Wang, Dabing Zhang

**Affiliations:** Key Laboratory of Animal Epidemiology and Zoonosis of Ministry of Agriculture, College of Veterinary Medicine, China Agricultural University, Beijing, China

**Keywords:** duck, Tembusu virus, attenuation, neutralizing antibody, envelope protein, stem region

## Abstract

Tembusu virus (TMUV) is a mosquito-borne flavivirus that most commonly affects adult breeder and layer ducks. However, a TMUV-caused neurological disease has also been found in ducklings below 7 weeks of age, highlighting the need to develop a safe vaccine for young ducklings. In this study, a plaque-purified PS TMUV strain was attenuated by serial passage in BHK-21 cells. Using 1-day-old Pekin ducklings as a model, the virus was confirmed to be attenuated sufficiently after 180 passages, whereas the neutralizing antibody response elicited by the 180th passage virus (PS180) was substantially impaired compared with PS. The findings suggest that sufficient attenuation results in loss of immunogenicity in the development of the live-attenuated TMUV vaccine. Comparative sequence analysis revealed that PS180 acquired one mutation (V41M) in prM and four mutations (T70A, Y176H, K313R, and F408L) in the envelope (E) protein. To identify the amino acid substitution(s) associated with loss of immunogenicity of PS180, we rescued parental viruses, rPS and rPS180, and produced mutant viruses, rPS180-M41V, rPS180-A70T, rPS180-H176Y, rPS180-R313K, rPS180-L408F, and rPS180-M5, which contained residue 41V in prM, residues 70T, 176Y, 313K, and 408F in E, and combination of the five residues, respectively, of PS in the backbone of the rPS180 genome. The neutralizing antibody response elicited by rPS180-L408F and rPS180-M5 was significantly higher than those by other mutant viruses and comparable to that by rPS. Furthermore, we produced mutant virus rPS-F408L, which contained residue 408L of PS180 in the backbone of the rPS genome. The F408L mutation conferred significantly decreased neutralizing antibody response to rPS-F408L, which was comparable to that elicited by rPS180. Based on homologous modeling, residue 408 was predicted to be located within the first helical domain of the stem region of the E protein (EH1). Together, these data demonstrate that a single mutation within the EH1 domain exerts a dramatical impact on the TMUV neutralizing antibody response. The present work may enhance our understanding of molecular basis of the TMUV neutralizing antibody response, and provides an important step for the development of a safe and efficient live-attenuated TMUV vaccine.

## Introduction

Tembusu virus (TMUV) is a mosquito-borne *Flavivirus* responsible for outbreaks of an infectious viral disease in ducks, originally known as duck hemorrhagic ovaritis (Cao et al., 2011). The disease is characterized by a sudden onset and quick spreading through the flock, a significant decrease in feed intake, a severe drop in egg production, and a degenerate ovary with hemorrhagic lesions (Cao et al., 2011; Su et al., 2011; Yan et al., 2011; Thontiravong et al., 2015). TMUV infection most commonly affects breeder and layer ducks during laying period. However, a TMUV-caused neurological disease has also been identified in both broiler and layer ducks below 7 weeks of age, resulting in signs of illness with ataxia, lameness, and paralysis (Yun et al., 2012a; Homonnay et al., 2014; Thontiravong et al., 2015; Liang et al., 2019). Although under field conditions flocks affected by TMUV do not show a significant increase in mortality, experimental infections with TMUV isolates result in deaths in ducklings below 2 weeks of age. Depending on the age of ducklings at the time of infection, the virulence of virus, and the dosage and the route of infection, the mortality varies considerably, ranging from 18 to 90%. Notably, the mortalities resulted from experimental infections are age dependent (Yun et al., 2012a; Sun X. Y. et al., 2014; Li et al., 2015; Lu et al., 2016; Liang et al., 2019). These investigations have raised concern over the threat of TMUV infection to ducklings, including breeder and layer ducks during the brood stage and commercial meat-type ducklings (Liang et al., 2019).

Tembusu virus-caused disease was first described in 2010 in China (Cao et al., 2011; Su et al., 2011; Yan et al., 2011). Subsequently, it was reported in Malaysia (Homonnay et al., 2014) and Thailand (Thontiravong et al., 2015). Since the emergence of the disease, different types of TMUV vaccine candidates have been developed in China, such as live-attenuated vaccines (Li et al., 2014; Sun L. et al., 2014; Wang et al., 2016; He et al., 2019; Huang et al., 2019; Zhang et al., 2020), inactivated vaccines (Lin et al., 2015; Zhang et al., 2017; Liu et al., 2018), subunit vaccines (Zhao et al., 2015; Ma et al., 2016), recombinant duck enteritis virus-, Newcastle disease virus-, and adenovirus-vectored vaccines (Chen et al., 2014; Zou et al., 2014, 2017; Sun et al., 2018; Tang et al., 2019), and DNA vaccines (Huang et al., 2018a, b; Tang et al., 2018). Among them, live-attenuated TMUV WFG36 (Wang et al., 2016) and FX2010 (Li et al., 2014) vaccines and inactivated TMUV HB vaccine (Liu et al., 2018) have been licensed to use in ducks in China. In 2012, our group also started a live-attenuated TMUV vaccine development project by using a plaque-purified PS TMUV strain as a starting material. Recent test of the neutralizing antibody response elicited by the 180th passage virus (PS180) revealed that the classical challenge of achieving an appropriate balance between sufficient attenuation and retention of immunogenicity has also been encountered in the development of the live-attenuated TMUV vaccine (Lv et al., 2019), as described previously for other viruses (Meng et al., 2014; Schmidt et al., 2019; Zhang et al., 2019).

More recently, we have reported the detection of neutralizing antibodies in sera of ducks infected by TMUV and vaccinated with the licensed attenuated TMUV WFG36 vaccine. The observation showed that both infection and vaccination elicit high levels of neutralizing antibody, and that neutralizing antibodies correlate with protection against challenge following vaccination (Lv et al., 2019). These data suggest that, like other flaviviruses (Pierson and Diamond, 2015; Diamond et al., 2008), humoral immune response plays the critical role in protection from TMUV. Furthermore, neutralizing antibody titers can be used as predictors of protection from infection, especially in the context of TMUV vaccine-induced protective immune response. Thus, the effect of attenuation on immunogenicity can be evaluated by detection of neutralizing antibodies in the development of a live-attenuated TMUV vaccine.

In common with other flaviviruses, TMUV possesses a single-stranded, positive-sense RNA genome (∼11 kb in size) that encodes three structural proteins [capsid (C), precursor of membrane/membrane (prM/M), and envelope (E)] and seven non-structural proteins (NS1, NS2A, NS2B, NS3, NS4A, NS4B, and NS5) (Yun et al., 2012a, b). Recent works with genetic engineering vaccine have shown that the E, prM, and C proteins of TMUV can induce protective immune responses (Chen et al., 2014; Zou et al., 2014, 2017; Zhao et al., 2015; Ma et al., 2016; Huang et al., 2018a, b; Sun et al., 2018; Tang et al., 2018). Nevertheless, data relating the molecular basis of TMUV vaccine-induced neutralizing antibody response is limited. In the case of flaviviruses, the E protein is the principal antigen that elicits neutralizing antibodies (Roehrig, 2003; Heinz and Stiasny, 2012). Although the prM and NS1 proteins also elicit protective antibody response, antibodies to the prM protein display limited neutralization potential (Vázquez et al., 2002; Beltramello et al., 2010; Dejnirattisai et al., 2010; de Alwis et al., 2011; Smith et al., 2012), and antibodies to the NS1 protein are non-neutralizing (Chung et al., 2007; Beatty et al., 2015). Therefore, identifying the amino acid changes in the E and prM proteins following passage is a key step toward understanding how attenuation results in loss of immunogenicity in the development of the live-attenuated TMUV vaccine.

In this study, we report the preparation of PS180 by serial passage of the plaque-purified PS TMUV strain in BHK-21 cells. Using 1-day-old Pekin ducklings as a model, the effect of attenuation on immunogenicity of the PS180 virus was investigated by comparing its neutralizing antibody response with that of the PS virus. To understand the molecular basis of attenuation-related loss of immunogenicity, we identified the amino acid mutations(s) responsible for the difference in neutralizing antibody response between PS180 and PS.

## Materials and Methods

### Cells and Virus

BHK-21 cells were cultured in T25 flasks in 5 ml of growth medium consisting of Dulbecco’s Modified Eagle Medium (DMEM) (Gibco, NY, United States) supplemented with 10% fetal calf serum (FCS) (Gibco, NY, United States), 100 U/ml penicillin, and 100 μg/ml streptomycin. The cells were incubated at 37°C. To develop a live-attenuated TMUV vaccine candidate, a plaque-purified PS virus was used as the initial inoculum. Information relating to the source and plaque-purification of the PS TMUV isolate was described previously (Liang et al., 2016). The rPS strain used in this study was rescued previously (Liang et al., 2016).

### Serial Passage of Virus in BHK-21 Cells

The PS virus was propagated in BHK-21 cells grown in T25 flasks as described previously (Lv et al., 2019). The infected cells were maintained in 5 ml of maintenance medium consisting of DMEM supplemented with 2% FCS, 100 U/ml penicillin, and 100 μg/ml streptomycin. The cells were incubated at 37°C and monitored daily for cytopathic effect (CPE). Cell cultures were freeze-thawed three times between 48 and 60 h post inoculation (p.i.). Following centrifugation at 10,000 × *g* for 1 min, supernatant was harvested for subsequent passage. During the passages, 1 ml of supernatant diluted 10- to 1000-fold in DMEM was used as inoculum. Each 10th passage was titrated, which were conducted by plaque assay as described recently (Lv et al., 2019). Viral titers are expressed as plaque forming unit (PFU)/ml. The passage viruses were stored at −80°C.

### Animals

Pekin ducklings were obtained from Peking duck breeding farm, Institute of Animal Sciences, Chinese Academy of Agricultural Sciences, Beijing, China. In the farm, TMUV-related disease had never been observed, and all flocks of breeders had never been vaccinated for TMUV. All ducklings used were confirmed to be free of TMUV infection and vaccination by testing antibodies in serum samples using a recently reported plaque reduction neutralization test (PRNT) for the detection of neutralizing antibodies against TMUV. In all animal experiments as described below, inoculation of ducklings was conducted at 1 day of age, and ducklings (*n* = 10) in each group were housed in a separate isolator.

Animal experiments were performed according to the Guide for the Care and Use of Laboratory Animals of the Ministry of Science and Technology of the People’s Republic of China. The protocols involving in animals were approved by the Animal Welfare and Ethical Censor Committee at CAU (CAU20171011-2).

### Test for Attenuation in Ducklings

The virulence of the 100th, 120th, 140th, 160th, and 180th passage viruses (PS100, PS120, PS140, PS160, and PS180) in ducklings were investigated. In every case, each of ducklings was inoculated intramuscularly (i.m.) with 0.2 ml (approximately 10^6^ PFU) of virus. An additional group of ducklings, inoculated i.m. with DMEM (0.2 ml/bird), served as a control. Ducklings were monitored daily for 7 days. The virulence of the passage virus in ducklings was evaluated on the basis of clinical signs, gross lesions, and weight gain.

### Passage of Attenuated Virus in Ducklings

The stability of PS160 and PS180 was tested by serial passage in ducklings. In each case, ducklings were divided into two groups: one group was inoculated i.m. with virus (approximately 5 × 10^6^ PFU/bird) and the other with DMEM (0.2 ml/bird). At 2 days p.i., five ducklings inoculated with virus were euthanized, and their spleens were collected and processed as 20% homogenate in phosphate-buffered saline (PBS). After centrifugation at 12,000 × *g* at 4°C for 15 min, the supernatant was harvested for subsequent passage. The remaining ducklings were monitored for additional 5 days. The virulence of the virus in ducklings was assessed as described above. Passage of the virus in ducklings was repeated for four times. Prior to passage, the presence of TMUV in the supernatant was confirmed by using a previously reported reverse transcription (RT)-PCR assay targeting the NS3 gene (Cao et al., 2011). The ducklings in the control group were treated identical to the group inoculated with virus.

### Test for Immunogenicity of PS180

Two experiments were conducted to evaluate the immunogenicity of PS180. In each experiment, ducklings were inoculated i.m. with PS180 (approximately 10^5^ PFU/bird) at 1 day of age. The plaque-purified PS virus was used for comparison. An additional group of ducklings inoculated with DMEM (0.2 ml/bird) served a control. In the first experiment, serum samples were collected from all ducklings at 7 days p.i. In the second experiment, sera were sampled from all ducklings at 1, 2, 3, and 4 weeks p.i. and tested for neutralizing antibodies against TMUV.

### Detection of Neutralizing Antibodies

Neutralizing antibodies in sera of TMUV-inoculated ducks was detected by using a previously reported PRNT, employing the 7th passage in BHK-21 cells of TMUV strain PS (PS7) as working virus (Lv et al., 2019). Briefly, 10-fold dilutions of the sera were mixed with an equal volume of diluted virus to give 40 PFU in 0.1 ml. After incubation at 37°C for 1 h, the virus-serum mixtures were inoculated onto BHK-21 cells. Following 1-h adsorption at 37°C, the plaque assay was conducted. The PRNT titer was calculated using the Kärber method and is expressed as 50% end point titer (neutralizing dose, ND_50_) as described recently (Lv et al., 2019).

### Genomic Sequencing

To identify sequence changes in the PS180 virus, the full-length genome sequence of the virus was determined and compared with that of the PS virus reported by Liang et al. (2016). Briefly, RNA was extracted from 250 μl of supernatant using a TRIpure Reagent (Aidlab, Beijing, China). cDNA was synthesized with a HiScript II Q RT SuperMix (Vazyme, Nanjing, China). Twelve overlapped fragments covering the entire genome were amplified using previously reported primers and conditions (Liang et al., 2016). The amplicons were purified using an EasyPure Quick Gel Extraction Kit (TransGen Biotech, Beijing, China) and sequenced commercially (Tianyi Huiyuan, Beijing, China). Sequence alignment was conducted using ClustalW^1^.

### Construction of Full-Length cDNAs for Parental and Mutant Viruses

The full-length cDNA of parental virus PS180 was constructed as described previously (Liang et al., 2016). Briefly, five overlapping fragments (designated A, B, C, D, and E) spanning the entire genome were amplified by RT-PCR with primers reported by Liang et al. (2016). T7 promoter and three non-templated G residues were introduced into the 5′ terminus of fragment A. The amplified A and B fragments were individually cloned into plasmid pClone-Blunt (Taihe Biotech, Beijing, China). Using the resulted pClone-Blunt-A and pClone-Blunt-B recombinant plasmids as templates, fragments A and B were individually amplified by PCR, and fused together by fusion PCR to generate fragment FI. The B, C, D, and E fragments were cloned in order into plasmid pBR322 using a ClonExpress II One Step Cloning Kit (Vazyme, Nanjing, China). The resulted pBR322-BCDE recombinant plasmid was used as template to produce fragment FII by PCR. The FI and FII fragments were assembled by fusion PCR to generate the full-length cDNA. The virus rescued from the full-length cDNA was designated rPS180.

To identify amino acids associated with loss of immunogenicity of PS180, PCR-based site-directed mutagenesis was performed to construct the full-length cDNAs of mutant viruses carrying residues of PS in the backbone of the rPS180 genome. Briefly, we targeted residue 41 in the prM protein and residues 70, 176, 313, and 408 in the E protein for mutagenesis on the basis of comparative sequence analysis. To introduce mutations into the rPS180 genome, fragment A was amplified from the pClone-Blunt-A recombinant plasmid with the primers shown in Table 1. This fragment A was used to construct the full-length cDNA by replacing fragment A described above. Using this strategy, the full-length cDNAs were constructed for six mutant viruses. They were rPS180-M41V, rPS180-A70T, rPS180-H176Y, rPS180-R313K, rPS180-L408F, and rPS180-M5, which carried residue Val^41^ in prM (prM-V41), residues Thr^70^ (E-70T), Tyr^176^ (E-176Y), Lys^313^ (E-313K), and Phe^408^ (E-408F) in E, and combination of the five residues, respectively, of the PS virus.

**TABLE 1:** Primers applied to introduce mutations into the prM and E proteins.

Primer^*a*^	Sequence (5′→3′)^*b*^	Mutation
prM41f	TGCTCTAGATGTGGGACTAATGTGTCAGGAT	M41V in prM of rPS180
prM41r	ATTAGTCCCACATCTAGAGCACGTACGACGC	
Env70f	AGACGTGACGACAGAATCCAGATGCCCAACC	A70T in E of rPS180
Env70r	CTGGATTCTGTCGTCACGTCTGACACTTTCG	
Env176f	AAGTCCCGTCTACACCGCTGAGATGGAGGAT	H176Y in E of rPS180
Env176r	TCAGCGGTGTAGACGGGACTTTTGGGCGTTA	
Env313f	TCCCTAGTGAAGAATCCTACCGACACTGGGC	R313K in E of rPS180
Env313r	GGTAGGATTCTTCACTAGGGAAAATGTGTTG	
Env408f	GAAAAGCTTTTACGTCAACACTCAAAGGAGC	L408F in E of rPS180
Env408r	GTGTTGACGTAAAAGCTTTTCCAATTGTGCT	
PSE408f	GAAAAGCTTTAACGTCAACACTCAAAGGAGCACAAAGGATGG	F408L in E of rPS
PSE408r	GTGTTGACGTTAAAGCTTTTCCAATTGTGCTCCCACTTCTAT	

*^a^f, forward primer; r, reverse primer. ^b^Nucleotides introduced by PCR are underlined.*

Similarly, we produced the full-length cDNA for mutant virus rPS-F408L, which carried residue Leu^408^ in E (E-408L) of PS180 in the backbone of the rPS genome. The full-length cDNA was generated using recombinant plasmids that were constructed previously for rescue of rPS (Liang et al., 2016). The primers used to introduce the F408L mutation are shown in Table 1.

### *In vitro* Transcription and Transfection

Each of the full-length cDNAs described above was transcribed with RNA polymerase by using an mMESSAGE mMACHINE T7 Kit (Ambion, Austin, TX, United States) according to the manufacturer’s instructions. The transcribed RNA was purified with lithium chloride provided by the kit. After quantitation with ultramicro nucleic acid protein concentration analyzer (Biodropsis BD-1000), 1 μg of the purified RNA was transfected into BHK-21 cells by using Opti-MEM I Reduced Serum Medium and Lipofectamine Messenger MAX Transfection Reagent (Invitrogen, Carlsbad, CA, United States), following the manufacturer’s instructions. The transfected cells were then incubated at 37°C in a 5% CO_2_ atmosphere. 10 h later, the medium was aspirated, cells were washed with PBS, and fresh maintenance medium was added. Incubation at 37°C in a 5% CO_2_ atmosphere continued. After 60 h, the cells were freeze-thawed once, followed by centrifugation at 4°C at 10,000 × *g* for 1 min. Supernatant was harvested for further passage in BHK-21 cells until a marked CPE was observed. In each passage, a 0.2-ml volume of supernatant was used as the inoculum. The rescued viruses were identified by genome sequencing as described above.

### Analysis of Growth Property

Confluent monolayers of BHK-21 cells (5 × 10^6^ cells) grown in T25 flasks were infected with virus at a multiplicity of infection (MOI) of 0.01 PFU/cell. After adsorption at 37°C for 1 h, the inoculum was aspirated and replaced with 5 ml of maintenance medium. To investigate the plaque phenotype of virus, samples (medium only) were collected at 60 h p.i. and diluted 1000-fold with DMEM. 0.2 ml of diluted virus was used in plaque assay. To assess the growth kinetics of virus, samples (medium only) were collected at different time points p.i., and viral titers were determined using plaque assay in BHK-21 cells.

### Duck Experiments for Mutant Viruses

Two animal experiments were conducted to investigate the virulence of the rPS180-based mutant viruses and the rPS-based mutant virus in ducklings and the neutralizing antibody response elicited by these viruses, respectively. In each experiment, Pekin ducklings were inoculated i.m. with virus-containing supernatant (10^5^ PFU/duckling). rPS180 and rPS were used for comparison. An additional group of ducklings inoculated i.m. with 0.2 ml of DMEM served as a control. The inoculated ducklings were monitored daily for 7 days. Before inoculation and at 1, 3, 5, and 7 days p.i., blood samples were collected from three ducks in each group for the detection of viremia. At 7 days p.i., sera were sampled from all ducks for the detection of neutralizing antibodies against TMUV.

### Quantitation of Viremia

Viremia of TMUV-inoculated ducks was determined by measurement of viral RNA levels in blood samples using a real-time quantitative PCR (RT-qPCR) assay. Briefly, RNA extraction and cDNA synthesis were conducted as described above. The reaction mixture (20 μl) contained 0.2 μl of cDNA, 0.4 μl of each of forward and reverse primers (Yu et al., 2012), and 10 μl of 2× AceQ qPCR SYBR Green Master Mix (Vazyme, Nanjing, China). qPCR was performed using the conditions reported (Yu et al., 2012).

### Homology Modeling and Sequence Alignment

To identify the structural element(s) in which one or more residues were considered responsible for the difference in neutralizing antibody response between PS180 and PS, the PS180 E protein was analyzed by homology modeling using the Phyre2 software (Kelley et al., 2015). Japanese encephalitis virus (JEV) E protein [Protein Data Bank (PDB) accession number 5WSN] was used as a structural template. The predicted structure was presented using the PyMOL software (Schrodinger, 2010).

To determine whether the immunogenicity-related residue(s) of PS180 and PS are conserved in other flaviviruses, representative viruses within the genus *Flavivirus* were aligned. Sequence alignment was performed using CLUASTALW (see text footnote 1), which were presented using the GeneDoc program (Nicholas et al., 1997). Flaviviruses used for comparison included the following: JEV, GenBank accession number LC095865; yellow fever virus (YFV), U073166; dengue virus 1 (DENV-1), KT438580; dengue virus 2 (DENV-2), KP757171; dengue virus 3 (DENV-3), JN036395; dengue virus 4 (DENV-4), KT452794; tick-borne encephalitis virus (TBEV), AF091020; West Nile virus (WNV), AF202541; Zika virus (ZIKV), KY131441; TMUV PS, KT876991; and TMUV MM1775, AB110494.

### Statistical Analysis

Viral titers, body weight, RNA copies, and neutralizing antibodies were expressed as means ± standard deviations (SD). The differences between groups were compared by analysis of variance using GraphPad Prism (version 5.0) software (GraphPad Software Inc., San Diego, CA, United States). A *P* < 0.05 value was considered statistically significant.

## Results

### Attenuation of the PS Virus by Serial Passage

**FIGURE 1:**
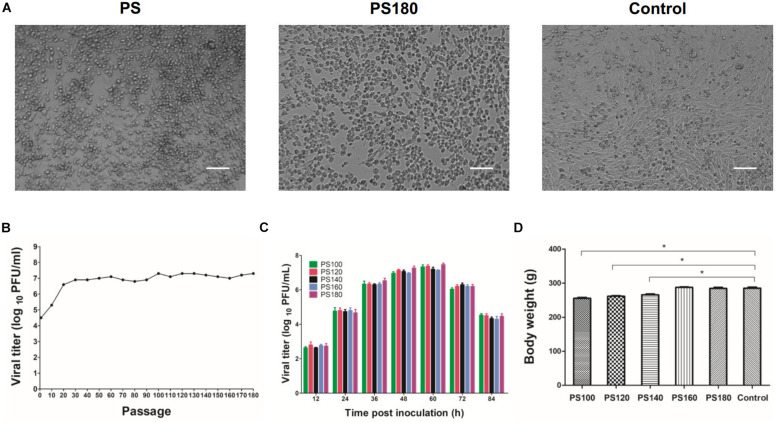
Attenuation of the PS strain of TMUV. **(A)** BHK-21 cells inoculated with the initial inoculum (PS) and the 180th passage of strain PS (PS180). Control, uninfected BHK-21 cells. Bar = 50 μm. **(B)** Serial passaging of PS in BHK-21 cells. Each 10th passage was titrated by using plaque assay. **(C)** Growth kinetics of PS100, PS120, PS140, PS160, and PS180 in BHK-21 cells. **(D)** Effect of PS100, PS120, PS140, PS160, and PS180 on weight gain of Pekin ducklings. The results shown are average body weight of 7-day-old ducklings inoculated 6 days previously with virus. *P < 0.05.

To develop a live-attenuated TMUV vaccine, the plaque-purified PS virus was passaged serially in BHK-21 cells. The passaged viruses grown well in the cells, with CPE being generally observed between 48 and 72 h p.i. A Marked CPE was produced at approximately 60 h p.i. from passages 100 to 180 (Figure 1A). In the first 20 passages, viral titer increased steadily with increasing passage level. In the subsequent 160 passage, viral titer plateaued with a small fluctuation between 10^6.8^ PFU/ml (passage 80) and 10^7.3^ PFU/ml (passages 100, 120, 130, and 180) (Figure 1B). Analysis of growth curves showed no difference in replication kinetics among the PS100, PS120, PS140, PS160, and PS180 viruses (Figure 1C). Together, these data suggest that after 20 passages the replication property of the plaque-purified PS TMUV strain in BHK-21 cells is no longer affected greatly by further attenuation.

PS100, PS120, and PS140 were still pathogenic for 1-day-old ducklings. These viruses had a slight impact on feed intake and weight gain of ducklings (Figure 1D). In a proportion of inoculated ducklings, signs of reluctance to walk and gross lesions of spleen enlargement were seen. Inoculation of ducklings with PS160 and PS180 was shown to be safe (Figure 1D). In the stability analysis, however, PS160 reverted to virulence within four duck passages. In the case of PS180, all the ducklings remained healthy without loss of weight gain and spleen enlargement throughout the observation period. These results suggest that the PS virus has been attenuated sufficiently after 180 passages.

### Immunogenicity of the Attenuated PS180 Strain

**FIGURE 2:**
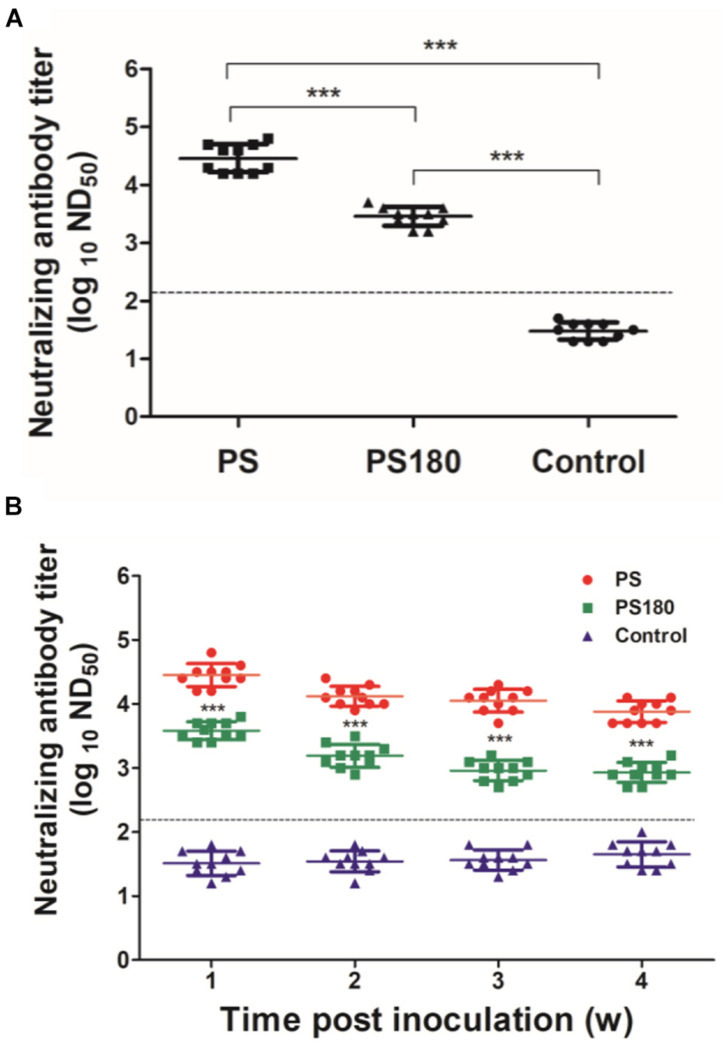
Influence of serial passages on immunogenicity of TMUV. Pekin ducklings were inoculated with virus at 1 day of age. Dotted line indicates cut-off value defined recently for negative and positive sera (Lv et al., 2019). **(A)** Neutralizing antibodies in sera of ducklings 7 days after inoculation with PS180 and PS. For each group, neutralizing antibodies in sera of ten ducklings were detected and shown. ***P < 0.001. **(B)** Duration of neutralizing antibodies in sera of ducklings after inoculation with PS180 and PS. Serum samples were collected at 1, 2, 3, and 4 weeks p.i. At each time point, serum samples of ten ducklings in each group were tested. ***, significant difference in mean neutralizing antibody between the PS180- and PS-inoculated groups (*P* < 0.001).

It has been shown previously that neutralizing antibody titers are commonly used as predictors of protection from infection, especially in the context of vaccine responses and immunity (Pierson et al., 2007). PRNT, which measures neutralizing antibodies, is the most virus-specific serological test and is generally considered the “gold standard” for the differentiation of flavivirus infections and the measurement of immunity to flaviviruses (Russell et al., 1967; Hombach et al., 2005. Putnak et al., 2008; Roehrig et al., 2008; Johnson et al., 2009; Rainwater-Lovett et al., 2012; Simões et al., 2012; Maeda and Maeda, 2013; Suthar et al., 2013; Lukman et al., 2016). Therefore, the immunogenicity of the attenuated PS180 strain was assessed by testing the serum samples using a previously reported PRNT for the detection neutralizing antibodies against TMUV (Lv et al., 2019). In this analysis, the neutralizing antibodies in sera of ducklings 7 days after inoculation with PS180 were detected and compared with those with PS. High ND_50_ titers (25119–63096) were detected in the 10 PS-inoculated ducks. In contrast, the ND_50_ titers detected in the 10 PS180-immunized ducks were generally lower, ranging from 1584 to 5012 (Figure 2A). There was significant difference between the PS180- and PS-inoculated groups and between the virus-inoculated groups and the control group (*P* < 0.05). Further analysis of the PS180 immunogenicity was performed by testing serum samples collected at different time points after inoculation with PS180 at 1 day of age. High ND_50_ titers were detected in sera collected at 1 (15848–63095), 2 (7943–25118), 3 (5011–15848), and 4 (5011–12589) weeks after infection with strain PS. The levels of ND_50_ titer detected in sera of ducklings inoculated with strain PS180 were generally low, ranging from 2511 to 6309 at 1 week p.i., from 794 to 2511 at 2 weeks p.i., from 501 to 1259 at 3 weeks p.i., and from 501 to 1259 at 4 weeks p.i. (Figure 2B). ND_50_ titers of below 1258, the minimum capable of providing adequate protection against challenge with virulent TMUV (Lv et al., 2019), were detected from 20, 60, and 80% of immunized ducklings at 2, 3, and 4 weeks p.i., respectively. These data indicate that sufficient attenuation of the PS TMUV strain confers a dramatically decreased neutralizing antibody response to the derived attenuated PS180 strain.

### Identification of Sequence Changes in PS180

To identify mutations associated with the difference in neutralizing antibody response between PS180 and PS, the full-length sequence of PS180 was determined and compared with that of PS previously reported by Liang et al. (2016). Comparative sequence analysis showed that a total of 25 nucleotide substitutions occurred in ORF of PS180. Of the nucleotide substitutions, 12 resulted in amino acid changes. They were Val^41^→Met^41^ (V41M) in prM; T70A, Y176H, K313R, and F408L in E; K347N in NS1; I232M in NS3; I49T in NS4B; and Q15K, K41R, S232G, and R543K in NS5 (A, Ala; H, His; R, Arg; N, Asn; I, Ile; Q, Gln; S, Ser; G, Gly). It has been previously shown that the flavivirus E protein is the principal antigen that elicits neutralizing antibodies (Roehrig, 2003; Heinz and Stiasny, 2012). Moreover, neutralizing antibodies that recognize prM protein have also been identified (Vázquez et al., 2002; Beltramello et al., 2010; Dejnirattisai et al., 2010; de Alwis et al., 2011; Smith et al., 2012). Therefore, the decreased immunogenicity of PS180 can be linked to one or more of the five mutations found in the prM and E proteins.

### Rescue of Parental and Mutant Viruses

**FIGURE 3:**
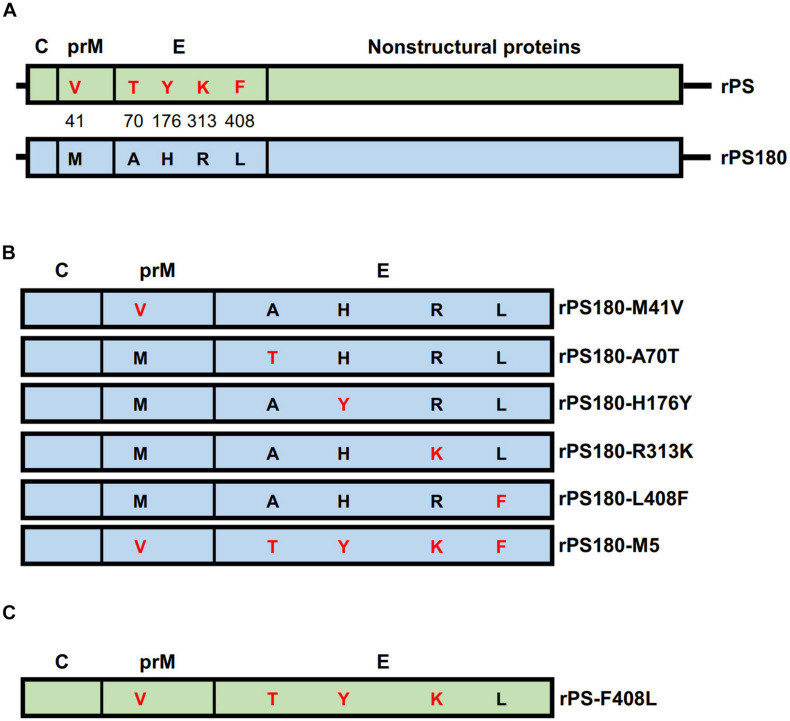
Construction of full-length cDNAs of TMUV. The genomic regions of parental backbone viruses rPS and rPS180 are represented with boxes and highlighted with light green and light blue, respectively. Proteins are labeled above the gene boxes. **(A)** Diagram of full-length cDNAs of rPS and rPS180. Amino acids in the prM and E proteins that differed between PS and PS180 are indicated with single letters in the gene boxes and highlighted with red (for PS) and black (for PS180). Their positions in the prM and E proteins are shown between the gene boxes. **(B)** Diagram of structural protein-encoding regions of mutant viruses carrying single mutations in the backbone of the rPS180 genome. **(C)** Diagram of structural protein-encoding region of a mutant virus carrying a single mutation in the backbone of the rPS genome.

**FIGURE 4:**
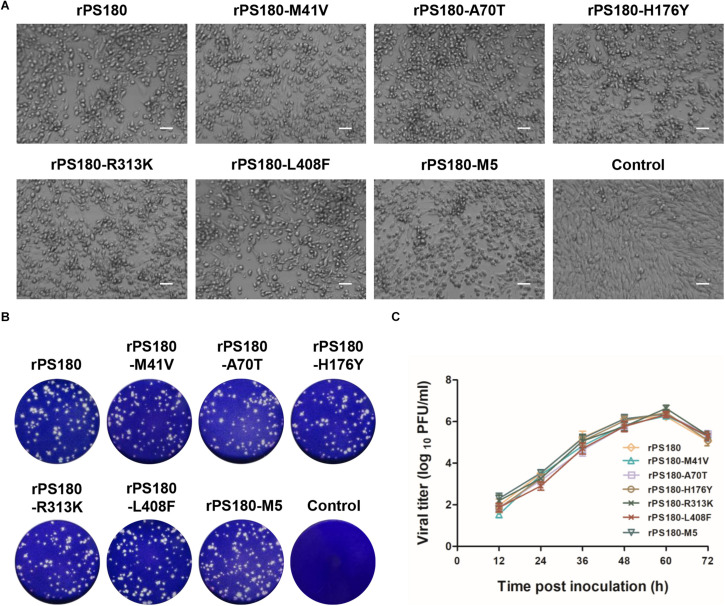
Growth properties of rPS180-based mutant viruses in BHK-21 cells. rPS180 was used for comparison. **(A)** CPE produced by the mutant viruses at 60 h p.i. Bar = 50 μm. **(B)** Plaques produced by the mutant viruses at 72 h p.i. **(C)** Growth kinetics of the mutant viruses.

To define the amino acids affecting the PS180 neutralizing antibody response, the five residues in the prM and E proteins that differed between PS180 and PS were targeted for PCR-based site-directed mutagenesis (Figure 3A). We produced full-length cDNAs for parental backbone virus rPS180 as well as mutant viruses rPS180-M41V, rPS180-A70T, rPS180-H176Y, rPS180-R313K, rPS180-L408F, and rPS180-M5, which contained a single mutation M41V in prM, single mutations A70T, H176Y, R313K, and L408F in E, and combination of the five mutations, respectively, in the background of the rPS180 genome (Figure 3B). Transfection of *in vitro* transcribed full-length RNAs into BHK-21 cells resulted in the rescue of the rPS180 virus and the mutant viruses. Following three passages, the rescued viruses produced a marked CPE, including cell rounding and detachment of cells from the surface of the plates (Figure 4A). Subsequently, we examined the growth properties of the rescued viruses in BHK-21 cells. The plaque sizes of all the rescued viruses were similar to one another, with a diameter of 1–2 mm (Figure 4B). The mutant viruses and their parental backbone virus rPS180 showed comparable replication kinetics (Figure 4C). These results indicate that the mutations in prM and E do not alter the growth properties of the mutant viruses in BHK-21 cells.

### Comparison of the Virulence of rPS, rPS180, and rPS180-Based Mutant Viruses in Ducklings

To investigate whether the five single mutations (M41V in prM and A70T, H176Y, R313K, and L408F in E) and combination of the mutations confer an enhanced virulence to the rPS180-based mutant viruses *in vivo*, 1-day-old Pekin ducklings were inoculated i.m. with rPS, rPS180, and the six rPS180-based mutant viruses. Like the rPS180-inoculated ducklings and the controls, all ducklings inoculated with the mutant viruses remained healthy throughout the observation period. In contrast, ducklings inoculated with rPS developed clinical signs typical of TMUV infection, such as decreased feed intake, ataxia, and paralysis. These data suggest that acquisition of the attenuated PS180 strain may not be attributed to the mutations at residue 41 in the prM protein and at residues 70, 176, 313, and 408 in the E protein.

**FIGURE 5:**
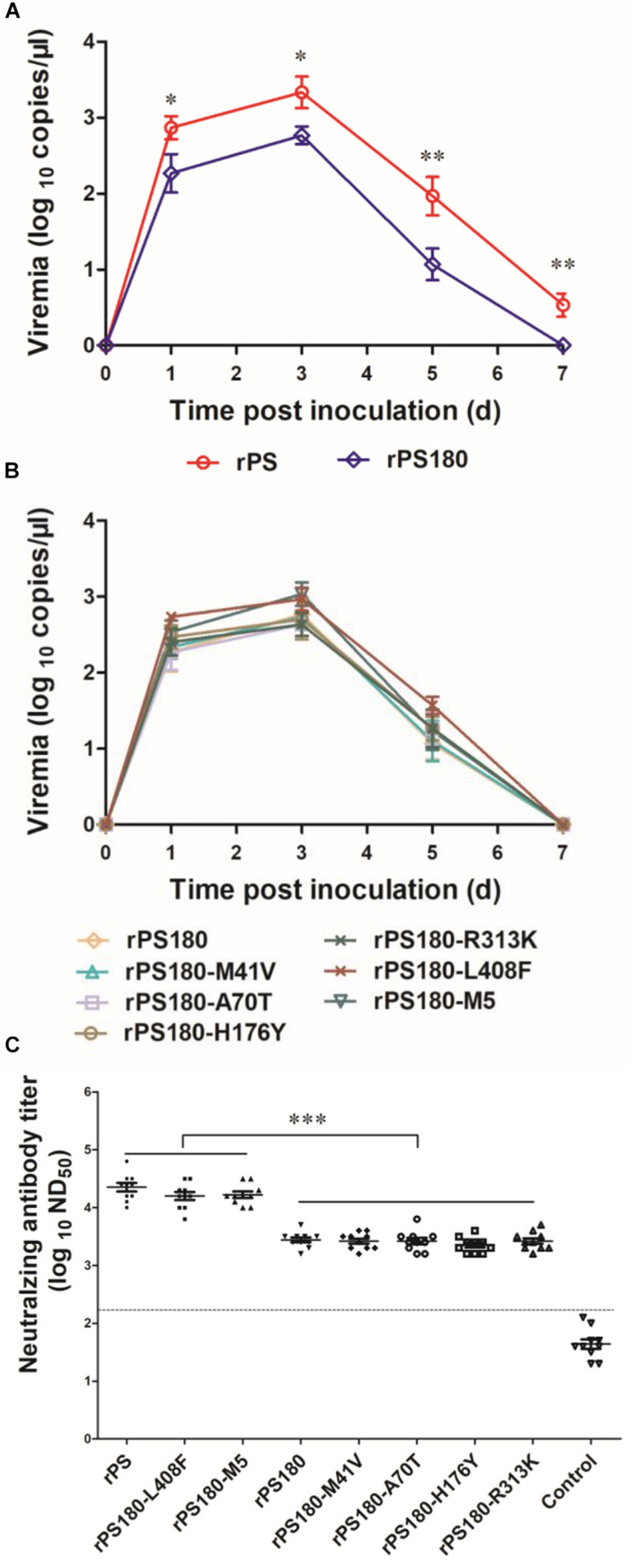
Influence of mutations of TMUV on induction of viremia and neutralizing antibodies in Pekin ducklings. The ducklings were inoculated with virus at 1 day of age. **(A)** Viremia of ducklings after inoculation with rPS and rPS180. Blood samples were collected at 1, 3, 5, and 7 days p.i. At each sampling point, RNA copies of three ducks in each group were tested and calculated as mean value ± S.D. *P < 0.05; **P < 0.01. **(B)** Viremia of ducklings after inoculation with rPS180 and rPS180-based mutant viruses. Blood samples were collected at 1, 3, 5, and 7 days p.i. At each sampling point, RNA copies of three ducks in each group were tested and calculated as mean value ± S.D. **(C)** Neutralizing antibodies in sera of ducklings 7 days after inoculation with rPS, rPS180, and rPS180-based mutant viruses. For each group, neutralizing antibodies of ten ducklings were tested and shown. Dotted line indicates cut-off value defined recently for negative and positive sera (Lv et al., 2019). ***P < 0.001.

Previous works have shown that viremia can be used to reflect the replication and virulence of TMUV *in vivo* (Yan et al., 2018). Thus, blood samples were collected from ducklings before and after inoculation with virus and tested for TMUV by using a previously reported RT-qPCR assay (Yu et al., 2012). Viremia were detectable in all virus-inoculated groups at 1, 3, and 5 days p.i. During this period, significantly lower levels of viremia were detected in the groups inoculated with rPS180 (Figure 5A), and the mutant viruses, as compared to the rPS-inoculated group (*P* < 0.05). Whereas there was no significant difference in viremia among the groups inoculated with rPS180 and the rPS180-based mutant viruses (Figure 5B). At 7 days p.i., no positives were detected from ducklings inoculated with rPS180 and the rPS180-based mutant viruses. In contrast, ducklings inoculated with rPS were tested positive (Figures 5A,B). These data indicate that strain rPS180 presents a significantly decreased viremic capacity relative to rPS, which might not be associated with the five mutations identified in the prM and E proteins.

### Effect of Mutations in prM and E Proteins on Immunogenicity of PS180

To define mutations responsible for the difference in neutralizing antibody response between PS180 and PS, we detected the neutralizing antibodies elicited by the rPS180-based mutant viruses at 7 days p.i., and compared with those of rPS and rPS180. High ND_50_ titers (6310–31622) were detected in sera of ducklings inoculated with the rPS180-M5 and rPS180-L408F mutant viruses, which were comparable to those (6310–63096) obtained from the rPS-inoculated ducklings. The ND_50_ titers detected in sera of ducklings inoculated with the rPS180-M41V, rPS180-A70T, rPS180-H176Y, and rPS180-R313K mutant viruses and the rPS180 virus were generally lower, ranging from 1584 to 5012 (Figure 5C). There was significant difference between the groups inoculated with rPS180-L408F, rPS180-M5, and rPS and the groups inoculated with rPS180 and other mutant viruses (*P* < 0.001). On average, we observed a five-fold increase in neutralization titers between the rPS180-L408F- and rPS180-inoculated groups. These findings indicate that only the E-L408F mutation significantly enhances the neutralizing antibody response elicited by rPS180.

### Effect of E-F408L Mutation on the PS Neutralizing Antibody Response

**FIGURE 6:**
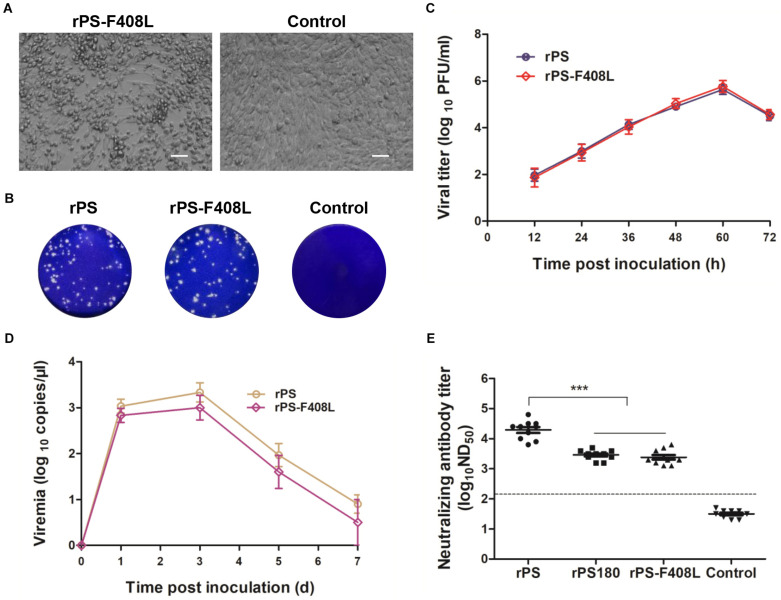
Influence of the F408L mutation in the E protein on growth properties *in vitro* and induction of viremia and neutralizing antibodies *in vivo*. **(A)** CPE developed by rPS-F408L in BHK-21 cells at 60 h p.i. Bar = 50 μm. **(B)** Plaques formed by rPS-F408L and rPS in BHK-21 cells at 72 h p.i. **(C)** Growth kinetics of rPS-F408L and rPS in BHK-21 cells. At each sampling time point, viral titers of three samples (medium only) were tested and calculated as mean ± S.D. **(D)** Viremia of Pekin duckling after inoculation with rPS-F408L and rPS. Ducklings were inoculated with virus at 1 day of age. Blood samples were collected at 1, 3, 5, and 7 days p.i. At each sampling time point, RNA copies of three ducklings in each group were tested and calculated as mean ± S.D. **(E)** Neutralizing antibodies in sera of 7-day-old Pekin ducklings inoculated 6 days previously with rPS-F408L, rPS, and rPS180. For each group, neutralizing antibodies in sera of ten ducklings were detected and shown. ***P < 0.001. Dotted line indicates cut-off value defined recently for negative and positive sera (Lv et al., 2019).

To further confirm the role of residue 408 in the E protein in determining the difference in neutralizing antibody response between PS180 and PS, we produced an additional mutant virus, rPS-F408L, which carried a single mutation (E-F408L) based on the backbone of the rPS genome (Figure 3C). The rPS-F408L mutant virus produced a marked CPE in BHK-21 cells at approximately 60 h p.i. (Figure 6A). The plaque size and growth kinetics of rPS-F408L were comparable to those of rPS (Figures 6B,C). Inoculation of 1-day-old Pekin ducklings with both rPS-F408L and rPS resulted in the development of clinical signs of illness, such as decrease in feed intake, ataxia, and paralysis. Using the TMUV-specific RT-qPCR assay (Yu et al., 2012), viremia was detectable in both rPS-F408L- and rPS-inoculated groups between 1 and 7 days p.i. (Figure 6D). There was no significant difference in viremia between the rPS-F408L- and rPS-inoculated groups (*P* > 0.05). Together, these results further confirm that residue 408 does not alter the growth property of TMUV in BHK-21 cells and the virulence of TMUV in ducklings.

We applied PRNT to detect neutralizing antibodies in sera of ducklings at 7 days p.i. High ND_50_ titers (6310–63096) were detected in the rPS-inoculated ducklings. In contrast, the ND_50_ titers detected in the rPS-F408L-inocualated ducklings were relatively low, ranging from 1258 to 6310, which were comparable to those (1584–5012) detected in the rPS180-inoculated ducklings (Figure 6E). Significant difference was observed between the rPS-inoculated group and the groups inoculated with rPS-F408L and rPS180 (*P* < 0.001). On average, we observed a six-fold decrease in neutralization titers between the rPS-F408L- and rPS-inoculated groups. These results indicate that the F408L mutation in the E protein of PS confers a dramatically decreased neutralizing antibody response to the rPS-based mutant virus. Together, these findings suggest that a single amino acid at residue 408 in the E protein is responsible for determining the difference in immunogenicity between PS180 and PS.

### Assessment of Genetic Stability of the L408F Mutation

To evaluate the genetic stability of the E-L408F mutation, mutant virus rPS180-L408F was passaged serially in BHK-21 cells. The 10th passage of rPS180-L408F (rPS180-L408F-P10) was subjected to full-length genome sequencing. Genome sequence comparison with rPS180-L408F showed that no mutations occurred in the genome sequence of rPS180-L408F-P10.

### Identification of Structural Element Containing Residue 408

**FIGURE 7:**
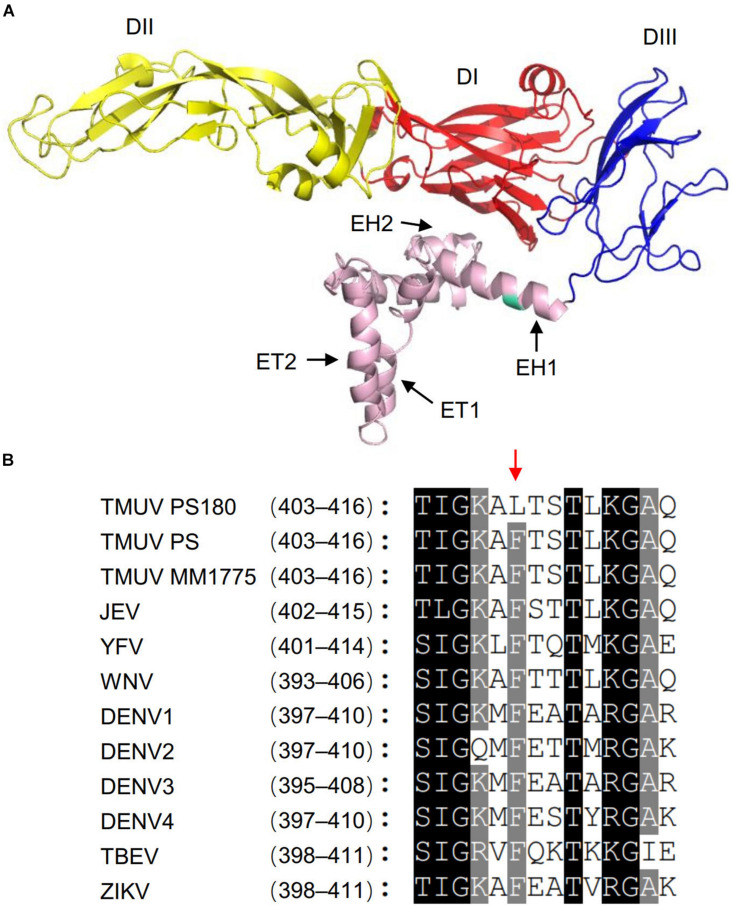
Identification of the PS180 E protein structural element containing residue 408L. **(A)** Homology modeling for the PS180 E protein. Color code for the E protein domains are as follows: domain I (DI), red; domain II (DII), yellow; and domain III (DIII), blue. EH1, α-helical domain 1; EH2, α-helical domain 2; ET1, transmembrane domain 1; ET2, transmembrane domain 2. Residue 408L is highlighted with green. **(B)** Amino acid sequence alignment of the PS180 EH1 domain with those of other flaviviruses. Numbers behind the virus name indicate positions of the EH1 domain in the E proteins of flaviviruses. Red arrow indicates residue 408 in the E proteins of the PS180, PS, and MM1755 strains of TMUV.

The flavivirus E protein comprises several structural elements. They are N-terminal ectodomain, which is composed of three distinct domains (DI, DII, and DIII); stem region, which contains two amphipathic α-helical domains (EH1 and EH2) and a stretch of conserved sequences; and two transmembrane domains (ET1 and EH2) (Allison et al., 1999; Lin et al., 2011; Heinz and Stiasny, 2012). To identify the structural element containing residue 408L, homology modeling was conducted for the E protein of PS180. The PS180 E-408L was found to be located within the EH1 domain (Figure 7A). Based on pairwise comparison of the PS180 EH1 sequence with those of other flaviviruses, the amino acid (F) at position 408 in the E proteins of PS and other TMUV isolates (e.g., MM1775) was highly conserved in other flaviviruses (Figure 7B). These results suggest that attenuation of the PS virus involves a single mutation at a highly conserved site within the EH1 domain.

## Discussion

In this study, we describe the attenuation of TMUV PS by serial passage in BHK-21 cells. We showed that viral titer increased approximately 1000-fold after 180 passages, indicating that the attenuated PS180 strain exhibits satisfactory growth property. On the basis of the safety and stability tests, the PS virus was shown to be sufficiently attenuated after 180 passages. An earlier work in our laboratory, which showed that vaccination of 1-day-old Pekin ducklings with 10^3^ PFU of PS180 offered adequate protection against an i.m. challenge of 10^5^ PFU of a virulent TMUV 7 days later, supports the view that PS180 displays satisfactory immunogenicity (Lv et al., 2019). Nevertheless, we have noted that, compared with PS, PS180 elicited dramatically lower levels of neutralizing antibody in Pekin ducklings 7 days after vaccination. Furthermore, the ND_50_ titers detected in 60–80% of ducklings 3–4 weeks after vaccination were lower than 1258, the minimum capable of providing adequate protection against challenge with virulent TMUV (Lv et al., 2019). Together, these results demonstrate that sufficient attenuation can result in loss of immunogenicity in the development of a live-attenuated TMUV vaccine.

On the basis that the E and prM proteins elicit flavivirus-specific neutralizing antibodies (Roehrig, 2003; Vázquez et al., 2002; Beltramello et al., 2010; Dejnirattisai et al., 2010; de Alwis et al., 2011; Heinz and Stiasny, 2012; Smith et al., 2012), the distinction in neutralizing antibody response between PS180 and PS that we have observed is likely to have important implications for elucidation of the molecular basis of attenuation-caused loss of immunogenicity. In the present study, therefore, we focused on the identification of amino acid substitution(s) that determined the difference in neutralizing antibody response between PS180 and PS by using a mutagenesis approach. We showed that the E-L408F mutation conferred significantly enhanced neutralizing antibody response to the rPS180-based mutant viruses, whereas the E-F408L back-mutation conferred dramatically decreased neutralizing antibody response to the rPS-based mutant virus. The fact that the rPS180-M5 and rPS180-L408F mutant viruses elicited very similar neutralizing antibody response supports the view that the five substitutions in the prM and E protein of PS180 do not exert synergistical effect on immunogenicity. Together these findings clearly demonstrate that the single mutation at residue 408 in the E protein can modulate neutralizing antibody response elicited by TMUV.

Epitopes in the E protein that are recognized by neutralizing antibodies reside in the ectodomain portion consisting of DI, DII, and DIII (Fibriansah and Lok, 2016; Dos Santos Franco et al., 2019; Li et al., 2019). Therefore, it is a surprise that a single amino acid substitution at residue 408 within the EH1 domain, downstream of DIII, dramatically reduced the PS180 neutralizing antibody response. Residue F408 is very conserved among flaviviruses, implying a functional role of the residue in the viral life cycle. Previous studies with DENV-4 and TBEV showed that the EH1 domain is associated with prM-E heterodimerization and plays an important role in increasing increases the stability of the E protein post fusion trimer, and modulating the trimer structure (Allison et al., 1999; Lin et al., 2011; Stiasny et al., 2013). We speculate that the mutation at residue 408 in the EH1 domain of TMUV may regulate conformational flexibility of E protein, and thereby contributes to the impact on immunogenicity of TMUV in ducks. However, how the mutation at residue 408 in the EH1 region regulates E protein conformational flexibility and how changes in E protein conformational flexibility affect immunogenicity of TMUV in ducks remain to be clarified.

In the investigation of the virulence of the mutant viruses in ducklings, all six mutant viruses constructed in the backbone of the rPS180 genome retained attenuated phenotype of their parental backbone virus, and rPS-F408L displayed a relatively low virulence similar to its parental backbone virus. The comparisons of viremic capacity between rPS180 and rPS, between rPS180-based mutant viruses and rPS180, and between rPS-F408L and rPS further confirmed the observations and suggested that rPS180 and rPS180-based mutant viruses can be more rapidly cleared from bloods of inoculated ducklings compared to rPS. These investigations suggest that the attenuation of PS might be attributed to one or more of the mutations (K347N in NS1, I232M in NS3, I49T in NS4B, and Q15K, K41R, S232G, and R543K in NS5) in the non-structural proteins, rather than the mutations (residue 41 in prM and residues 70, 176, 313, and 408 in E) in the structural proteins. We cannot conclude that the present observations are inconsistent with earlier findings of other workers (Yan et al., 2018; Sun et al., 2020), who reported that the E protein plays a critical role in determining the pathogenicity of TMUV in ducks. Our virus used for attenuation has undergone 11 passages, including five passages in embryonated chicken embryos, three passages in BHK-21 cells, three-rounds of plaque purification, and one passage in BHK-21 for viral stock preparation (Liang et al., 2016). We could not compare the virulence of the plaque-purified PS virus with its original field isolate. Nevertheless, compared with another field isolate (Y), which resulted in 80% mortality in Pekin ducklings following i.m. infection at 2 days of age (Liang et al., 2019), the virulence of the rPS virus in 1-day-old ducklings investigated in this study is extremely lower. The distinction in virulence between the rPS and Y strains suggests that the initial inoculum PS has been attenuated substantially, which might be attributed to mutation(s) in the E protein. Interestingly, the E proteins of both PS and PS180 strains contain two previously identified pathogenicity-related residues, 165S which was shown to be responsible for the high tissue tropism and transmissibility of FX2010 by comparing with mosquito-origin MM1775 (Yan et al., 2018), and 367T which was considered crucial for the high pathogenicity of JXSP by comparing the 310th passage virus of JXSP (JXSP-P310) (Sun et al., 2020). This suggests that the two residues do not contribute to the difference in virulence between the PS and PS180 strains. Similarly, both FX2010 and MM1775 contain Env 367T; both JXSP and JXSP-P310 contain Env 156S. Presumedly, virulence-related residues identified by comparison of two TMUV strains might be strain-specific. These findings will stimulate further studies on the molecular basis of TMUV virulence.

## Conclusion

Using the plaque-purified PS TMUV strain as a starting material, a sufficiently attenuated PS180 strain was produced after serial passage in BHK-21 cells. However, the neutralizing antibody response elicited by the attenuated virus was substantially impaired due to a single mutation at residue 408 (F408L) in the E protein. Interestingly, the E-L408F mutation significantly enhanced neutralizing antibody response, while did not alter the attenuated phenotype. Therefore, the resulted rPS180-L408F variant, which carried E-408F of PS in the backbone of the rPS180 genome, has potential for developing a recombinant TMUV live-attenuated vaccine. The present work may enhance our understanding of molecular basis of the TMUV neutralizing antibody response, and provides a new approach for the development of a safe and efficient live-attenuated TMUV vaccine.

## Data Availability Statement

The datasets presented in this study can be found in online repositories. The names of the repository/repositories and accession number(s) can be found in the article/Supplementary Material.

## Ethics Statement

The animal study was reviewed and approved by Animal Welfare and Ethical Censor Committee at CAU (CAU20171011-2).

## Author Contributions

DZ, XW, and JL designed the study and wrote the manuscript. JL, XL, and SC performed the experiments. LY and SQ participated in data analysis. RM, BY, and CF participated in animal experiments. All authors contributed to the article and approved the submitted version.

## Conflict of Interest

The authors declare that the research was conducted in the absence of any commercial or financial relationships that could be construed as a potential conflict of interest.

## Footnotes

1 https://www.genome.jp/tools-bin/clustalw
