# Supplementary material for: The Neutralizing Antibody Response Elicited by Tembusu Virus Is Affected Dramatically by a Single Mutation in the Stem Region of the Envelope Protein
Source: Front Microbiol. 2020 Oct 22;11:585194. doi: 10.3389/fmicb.2020.585194 (PMC7642334; doi:10.3389/fmicb.2020.585194)
Supplement: Supplementary file 1 [file Table_1.DOCX]

| **SUPPLEMENTARY TABLE S1**│Sequence changes between PS and PS180 | | | | | | | |
| --- | --- | --- | --- | --- | --- | --- | --- |
| Gene | Nucleotide substitution | | |  | Amino acid substitution | | |
|  | Position^a^ | PS | PS180 |  | Position^b^ | PS | PS180 |
| prM | 121 | GUG | AUG |  | 41 | V | M |
| E | 208 | ACA | GCA |  | 70 | T | A |
|  | 526 | UAC | CAC |  | 176 | Y | H |
|  | 938 | AAG | AGG |  | 313 | K | R |
|  | 1224 | UUU | UUA |  | 408 | F | L |
| NS1 | 1026 | AAA | AAC |  | 347 | K | N |
| NS3 | 696 | AUA | AUG |  | 232 | I | M |
| NS4B | 593 | AUA | ACA |  | 49 | I | T |
| NS5 | 43 | CAG | AAG |  | 15 | Q | K |
|  | 122 | AAG | AGG |  | 41 | K | R |
|  | 694 | AGU | GGU |  | 232 | S | G |
|  | 1628 | AGA | AAA |  | 543 | R | K |
| ^a^Nucleotide position in each gene.  ^b^Amino acid position in each protein. | | | | | | | |
